# A water-cooled monochromator for the B16 Test beamline at the Diamond Light Source: capabilities and performance characterization

**DOI:** 10.1107/S1600577518014662

**Published:** 2019-01-01

**Authors:** Igor P. Dolbnya, Kawal J. S. Sawhney, Stewart M. Scott, Andy J. Dent, Giannantonio Cibin, Geoff M. Preece, Ulrik K. Pedersen, Jon Kelly, Paul Murray

**Affiliations:** a Diamond Light Source, Harwell Science and Innovation Campus, Didcot, Oxfordshire OX11 0DE, UK; b Instrument Design Technology Ltd, Unit 2 Turnstone Business Park, Mulberry Avenue, Widnes WA8 0WN, UK

**Keywords:** X-ray optics, X-ray monochromators, synchrotron radiation

## Abstract

Systematic studies of the performance of a water-cooled X-ray monochromator, designed and built for the B16 Test beamline at the Diamond Light Source, UK, are presented. A technical description of the monochromator is given and the results of commissioning measurements are discussed. Overall, the monochromator satisfies the original specifications well and meets all the major requirements of the versatile beamline. Following its successful implementation on B16, the basic monochromator design has been reproduced and adapted on other Diamond Light Source beamlines, including B18 and B21.

## Abstract 2

Systematic studies of the performance of a water-cooled X-ray monochromator, designed and built for the B16 Test beamline at Diamond Light Source, UK, are presented.

## 1. Introduction   

**Figure 1:**
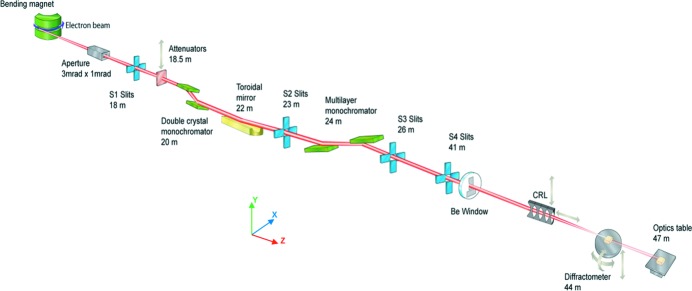
Schematic of the B16 Test beamline at the Diamond Light Source synchrotron.

Monochromators are key components of modern synchrotron radiation beamlines that come in many different types depending on the application of each particular beamline and photon energy range at which it operates: infrared, vacuum ultraviolet, soft or hard X-rays (Matsushita, 1983 ▸). A water-cooled monochromator has been designed and built for the B16 Test beamline at the Diamond Light Source (DLS), UK (Sawhney *et al.*, 2010 ▸). A schematic of the beamline is shown in Fig. 1 ▸. The device consists of a double-crystal monochromator (DCM)^1^, based on a pair of separate Si(111) crystals, and a Si(311) channel-cut crystal. In total, the monochromator covers an X-ray photon energy range from ∼4 keV (Be window cut-off) to ∼45 keV (4–20 keV with the DCM and 6–45 keV with the channel-cut). The measured photon flux delivered by the monochromator at typical beamline distances of 45–46 m is ∼2 × 10^9^ photons s^−1^ mm^−2^ (300 mA)^−1^ (0.1% bandwidth)^−1^ (with the DCM) and ∼2.5 × 10^8^ photons s^−1^ mm^−2^ (300 mA)^−1^ (0.1% bandwidth)^−1^ (with the channel-cut) at 10 keV.

## 2. Design description and principle components   

A wide range of different water and cryogenically cooled monochromators have been designed and built over the last two to three decades for applications with synchrotron radiation sources. In each case, the design is dictated by specific requirements of a particular beamline or experimental technique, including energy range, energy resolution, speed, beam stability and beam quality (Wahl *et al.*, 1992 ▸; Freund *et al.*, 1997 ▸; Schulte-Schrepping *et al.*, 1998 ▸; Suortti *et al.*, 2000 ▸; Zhang *et al.*, 2001 ▸).

**Figure 2:**
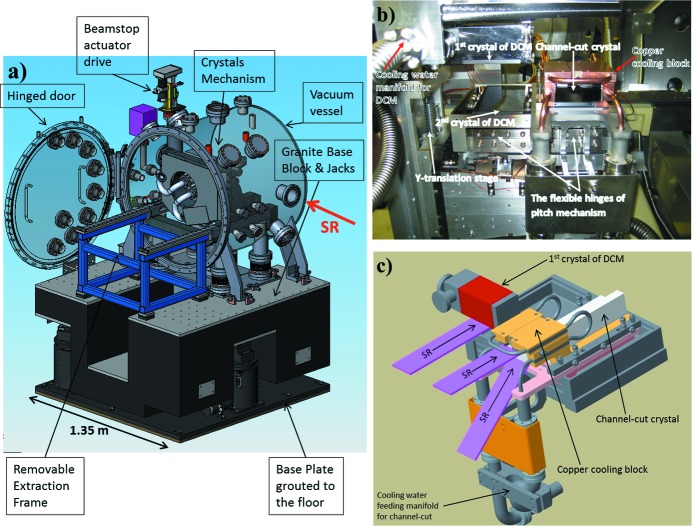
(*a*) Schematic of the whole monochromator assembly, (*b*) photograph of the monochromator crystal cage (shown at nearly zero Bragg angle) and (*c*) schematic representation of the crystal arrangement (the second crystal of the DCM is not shown) where the purple stripes represent the incoming X-ray beams under different incidence angles/Bragg rotations.

Our design follows a traditional approach where the centre of common Bragg rotation coincides with the surface of the first crystal of the DCM, or the first lamella of the channel-cut crystal when the latter is in use. The monochromator consists of a single rotary table (Bragg axis) that supports the water-cooled first crystal, the second crystal cage of the DCM and the channel-cut crystal assemblies. The monochromator is designed for bounce down operation. The design concept of the DCM is based upon using a sufficiently long 150 mm second crystal that allows the beam, reflected from the first crystal, to move along the second crystal as the energy is changed so that no longitudinal translation is required. The first crystal of the DCM is directly water cooled and has no water-to-vacuum joints. The second crystal of the DCM is not cooled and remains at room temperature. The Si(311) channel-cut crystal is indirectly water cooled via the copper block attached directly to the back of its first lamella, which receives the primary incoming ‘white’ beam. The second lamella of channel-cut is also 150 mm long. Fig. 2 ▸ shows the arrangement of the crystals inside the monochromator vacuum vessel.

The DCM provides a fixed-exit beam offset of 25 mm. It is realized by changing the gap separating the first and second crystals (*Y*-translation on the second crystal) accordingly while changing the photon energy (the Bragg angle). The position of the first crystal of the DCM (or the first lamella of the channel-cut) is fixed with respect to the incoming beam. The beam offset with the channel-cut monochromator is ∼20 mm and varies slightly over the energy range specified. The channel-cut crystal is mounted on the same bed plate of the crystal cage as the second crystal of the DCM. This arrangement provides all angular (roll and pitch) and positional motions necessary for operation of the channel-cut crystal. Switching between the DCM and channel-cut monochromators is accomplished by a simple transverse translation of the entire crystal assembly across the beam inside the vacuum vessel (X-translation) and by adjusting the vertical position of the channel-cut crystal with respect to the incoming beam. The whole monochromator vacuum vessel can also be moved vertically so that the ‘white’ beam can be passed through to the beamline (with the gap between first and second crystals of the DCM widely open).

The monochromator crystal assembly is mounted on a Newport in-vacuum rotary table (rotation speed: 0.4° s^−1^) that is driven in closed-loop operation using a custom encoder assembly comprising four Renishaw readheads located at the rear of the rotary table. The encoder scale is a Renishaw RESR ring using RGH20 readheads with 1000× interpolation. The encoder averaging is performed every servo-cycle by a Delta Tau PMAC controller. The energy axis calculation is also performed by the PMAC using inverse kinematics within a motion program. The four readheads are positioned 90° apart which cancels out the dominant axis eccentricity errors giving better accuracy and reproducibility.

The cage for the second crystal of the DCM and the channel-cut crystal is supported by aluminium mounts and incorporates the pitch and roll adjustments. The unit uses stainless steel blade springs to create backlash-free hinges. The wide blade spring design reduces problems associated with hinge misalignment. The pitch adjusting mechanism incorporates a high-stiffness 60 µm-range Jena piezo actuator, in line with the stepper drive, for high-frequency closed-loop operation. The coarse pitch and roll actuator drives employ a common design using a Phytron stepper motor/gearbox with a Schneeberger miniature ball screw. The linear drives are preloaded by stainless steel extension springs.

The internal mechanism is mounted on a multi-carriage, high-stiffness X-translation motorized slide. The whole monochromator is supported on a ∼2000 kg synthetic granite block which can be aligned using three kinematically mounted vertical jacks. These three jacks provide high stability with the functionality of motorized vertical *Y*-motion to align the monochromator with respect to the incoming beam, plus the ability to adjust the pitch and roll of the entire instrument.

A summary of the motorized degrees of motions of the monochromator is given in Table 1 ▸.

The monochromator was specified to accept 3 mrad × 0.16 mrad of horizontal and vertical angular beam divergence, respectively, from the B16 dipole magnet (1.4 T) radiation source, resulting in a total incident beam power of ∼130 W (with the DLS storage ring operating initially at 500 mA of electron beam current). The 0.16 mrad vertical angular divergence is dictated by the acceptance of the toroidal mirror that follows the monochromator on the B16 beamline.

The requirement to remove the induced heat load on the crystals and maintain the transmission efficiency throughout the crystals is crucial. Preliminary finite element analysis (FEA) studies were undertaken for various designs of water-cooled Si crystals, including indirectly side-cooled and directly cooled schemes. The side-cooled geometry was shown to be inadequate in this beamline application because the angular slope errors induced by heat loading on the crystal surface are unacceptably large. Therefore, for the DCM a directly water-cooled first-crystal scheme was chosen. The novel cooling design was optimized to provide the highest performance possible, in a compact assembly, over the wide energy range of 2–20 keV specified for the DCM.

**Figure 3:**
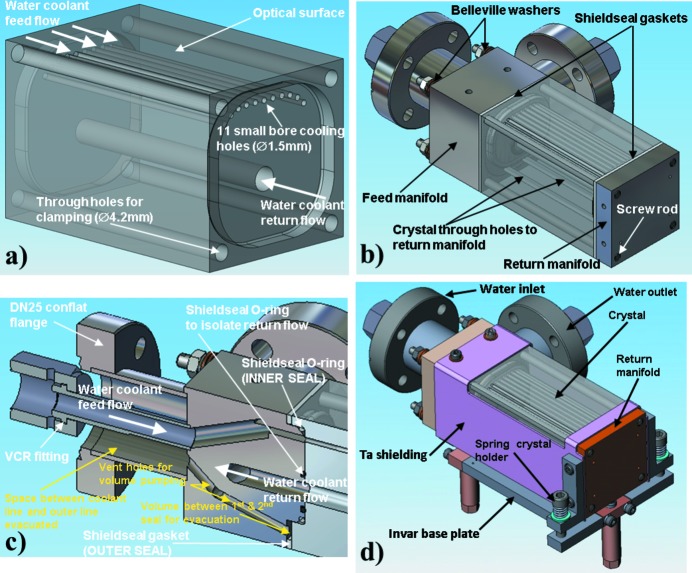
Design of the first crystal of the DCM: (*a*) the multi-channel small bore cooling scheme [crystal size: 44 mm (long) × 43 mm (deep) × 73 mm (wide)], (*b*) the clamped crystal arrangement, (*c*) the guard vacuum feed manifold and (*d*) the whole crystal mount.

Fig. 3(*a*) ▸ shows the design of the directly water-cooled first Si(111) crystal of the DCM with 11 small bore holes (1.5 mm diameter) drilled through the width of crystal in a curved pattern close to the crystal optical surface (recessed by 6.5 mm in its central part), through which the water flow is applied. This design gives sufficient space for two mechanical seals with Invar manifolds to be clamped to both sides of the crystal to feed and return the water coolant, as shown in Fig. 3(*b*) ▸. Invar was chosen because its expansion coefficient is close to that of silicon. For the seals, Shieldseal 641 O-rings and gaskets were used [Fig. 3(*c*) ▸]. Shieldseal 641 is a soft-grade fluoro­elastomer whose material properties include excellent radiation resistance and high-temperature stability.

The crystal sub-assembly is clamped together using four screw rods that are fed through the width of the crystal and screwed into the return manifold. The feed manifold assembly is then fed onto the screw rods and Belleville washers are used to apply the spring tension for the clamping nuts. To provide further leak protection, the void between the inner and outer seals can be pumped out as depicted in Fig. 3(*c*) ▸, forming the protective guard vacuum in the feed and return manifolds. Three springs are used to hold the crystal holder to the mount; two positioned either side of the flat fitting and one positioned between the fittings below the Invar base plate [Fig. 3(*d*) ▸].

Details of the FEA studies of the first crystal of the DCM performance under a specified heat load are given in Appendix *A* [App appa].

The water-cooling circuit to the first crystal of the DCM is protected by a guard vacuum. The water-return manifolds and water delivery pipes have an inner space where a moderate guard vacuum (∼10^−3^ mbar) is maintained by a roughing pump and this vacuum space is interlocked. Thus, the beamline vacuum integrity is guaranteed by a guard ring-and-gasket system to avoid the presence of water near to the main DCM vacuum (∼10^−8^ mbar) joints. The beamline vacuum control system constantly monitors the pressure and gas content of this inner guard space in order to detect any possible water leaks. In case of an accident, the control system would close the main beamline port shutter and stop the water chiller.

The channel-cut Si(311) crystal is cooled indirectly by a water-cooled split copper block attached to its first lamella [see Fig. 2(*b*) ▸] using the same water circuitry as the DCM. The crystal is adhered to the copper block using an intermediate layer of Wood’s metal eutectic alloy (melting point 70°C) that was first melted and then slowly solidified during the process of crystal installation. This method of mounting provided the strain-free clamping of the first channel-cut crystal lamella to the copper block and also an efficient and uniform thermal conductance between them.

**Figure 4:**
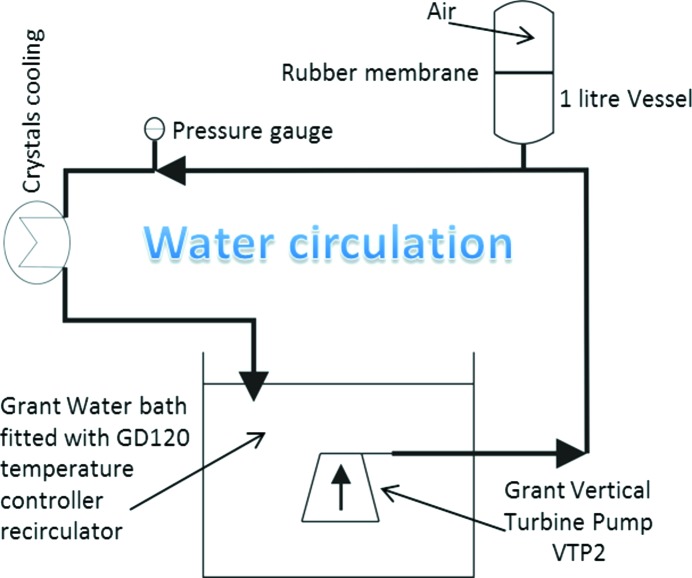
Schematic of the water-pressure pulse reduction chamber and chiller.

Some modifications to the monochromator water circuitry were made at a later stage to improve the overall stability and vibrational performance of the chiller (Grant Instruments Ltd, GR150 with an isolated vertical turbine pump and 5 l water capacity) and roughing pump. The pump and chiller were relocated onto anti-vibration feet and mounted on a large frame bolted to the floor of the beamline optics hutch. In addition, a pulse reduction chamber was installed on the water system, which absorbs any water pressure oscillations that could be transmitted to the first crystal of the DCM (see Fig. 4 ▸). This consists of a 1 l water buffer vessel separated from an isolated air chamber by a 10 mm-thick rubber membrane that acts as a shock absorber.

## 3. Performance   

### 3.1. Beam stability measurements   

**Figure 5:**
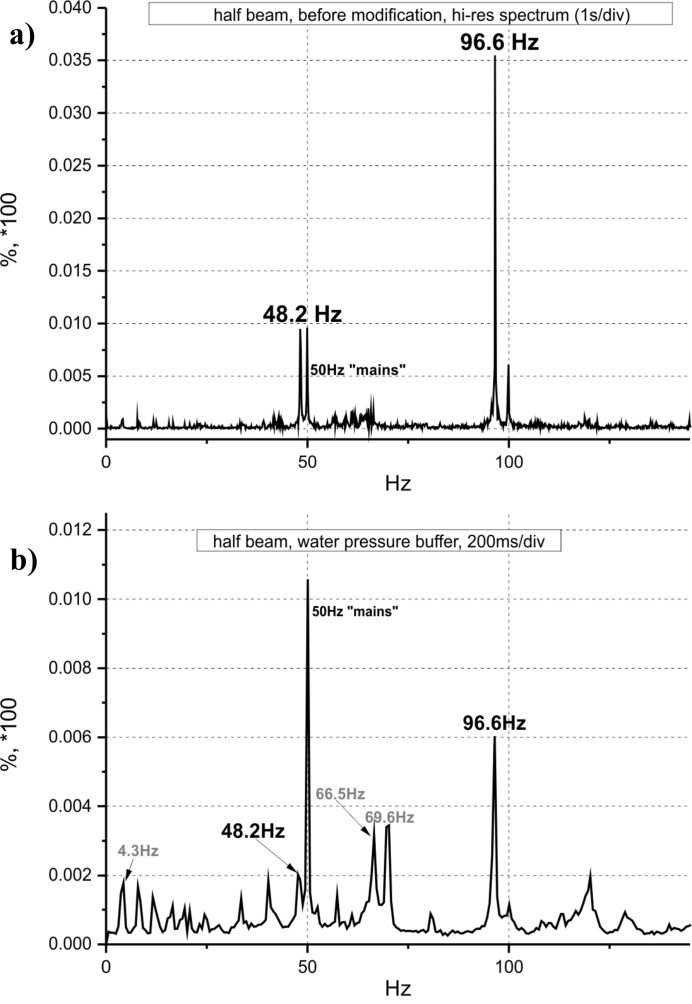
Beam vibration measurements shown as power spectra (*a*) before and (*b*) after modifications were made to the water chiller and the guard vacuum roughing pump.

Several tests have been performed to measure beam instabilities on the B16 beamline. Most of the measurements have been carried out with a LeCroy WaveRunner 64Xi oscilloscope using the built-in device functionalities, including the direct measurement of power spectra (fast Fourier transforms) of various signals coming from different point detectors in the beamline. A passivated implanted planar silicon (PIPS) photodiode (Canberra PD300-17-500AB) detector was used for measurements. The signal from the photodiode was amplified by a Keithley 428 current amplifier and the beam intensity was measured at ∼43.6 m from the bending magnet source (23.6 m from the monochromator). The DCM energy was set to 15 keV, a beam height of 200 µm was defined by vertical slits mounted just in front of the diode and the beam was halved by using a 1 mm-thick tantalum horizontal single blade attached directly to the diode face and adjusting the vertical position of the diode with respect to the beam. The size of the beamline primary vertical slits (located at 18.6 m from the radiation source) was also set to 200 µm. This arrangement allowed measurement of any possible vertical movements or vibrations of the beam. The major concern was low-frequency instabilities so the measured characteristic frequency range was restricted to ∼200 Hz. Fig. 5 ▸ shows the beam vibration data measured before and after modifications were made to the water chiller and roughing pump system as described above.

As seen in Fig. 5 ▸, the implementation of a water pressure buffer system significantly reduced vibrations at dominant frequencies that were observed at 48.2 and 96.6 Hz, from ∼1% (at 48.2 Hz) and 3.55% (at 96.6 Hz) to 0.2% and 0.6%, respectively. This corresponds to an acceptable beam migration of 100 µm × 0.6% = 0.6 µm (at 96.6 Hz) at the potential sample position. In terms of angular pitch vibration of the monochromator, this is equivalent to 0.6 × 10^−6^/23.6 = 0.0254 µrad, which is significantly less than the width of the rocking curve of the monochromator crystals at any energy.

The 50 Hz signal is always present due to electrical noise from the mains power supplies and not taken into consideration. There are some other frequencies that became more apparent after the water supply stabilization at 4.3, 7.94, 11.6, 16.5, 33.6, 40.3, 66.5 and 69.6 Hz; however, these are all below 0.4% and negligible.

**Figure 6:**
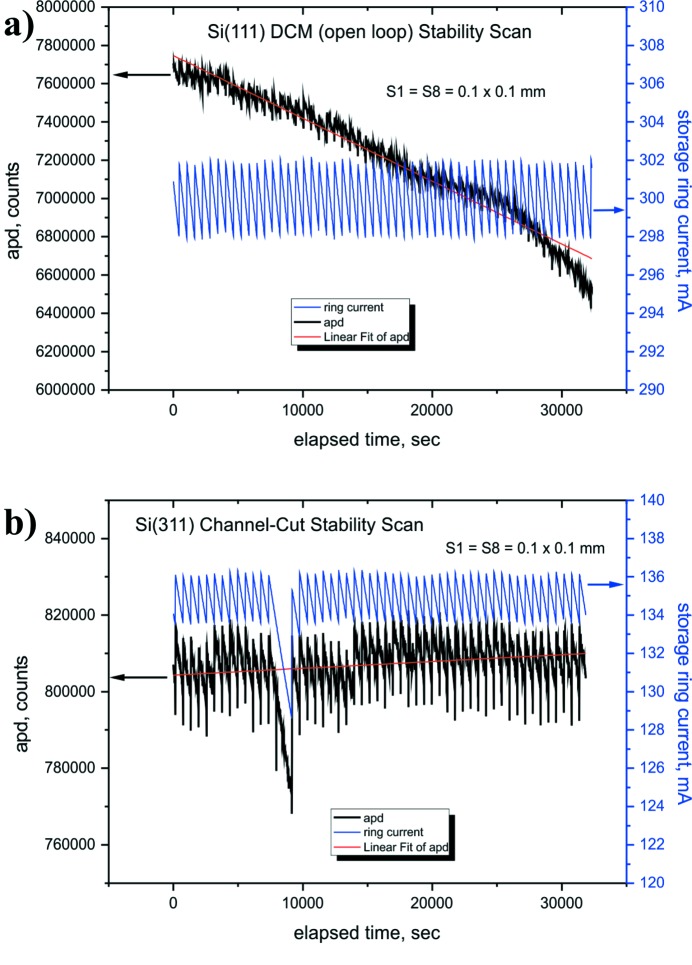
Long duration scans with data points taken every 10 s for the (*a*) DCM and (*b*) channel-cut monochromators at 15 keV. The electron beam top-up events are obvious. No closed-loop feedback was used on the DCM.

Long-term beam stability measurements were also undertaken and Fig. 6 ▸ shows typical data taken over a period of a few hours with the DCM and channel-cut. The measurements were taken every 10 s using an avalanche photodiode counting detector with the beamline primary slits and pre-detector slits (the latter at ∼47 m from the source) both set to 0.1 mm × 0.1 mm. The DLS storage ring operated in a top-up filling mode.

As may be seen, the measurements taken with the DCM [Fig. 6(*a*) ▸] show a slow variation of beam intensity over a period of a few hours, indicating a constant beam drift past the narrow pre-detector slits. For the particular time scan shown in Fig. 6(*a*) ▸, this drift amounted to ∼15% and the major component of this is likely to be the slow adjustment of the second crystal pitch because no variation of the electron beam orbit was observed during the measurements as indicated by two X-ray beam position monitors (XBPMs) installed in the beamline front-end and separated by a distance of 6 m from each other.

This slow drift of the beam intensity could adversely affect some measurements and certain experiments undertaken on a beamline, especially those performed with focused beams and which involve measurement times of several hours. However, this can often be normalized with an incident beam intensity monitor, which is typically an ionization chamber measuring the beam intensity near the sample position and *after* the final collimation and/or focusing.

The comparable stability measurements with the channel-cut crystal [Fig. 6(*b*) ▸] show stable beam behaviour, as may be expected.

### 3.2. Energy stability   

**Figure 7:**
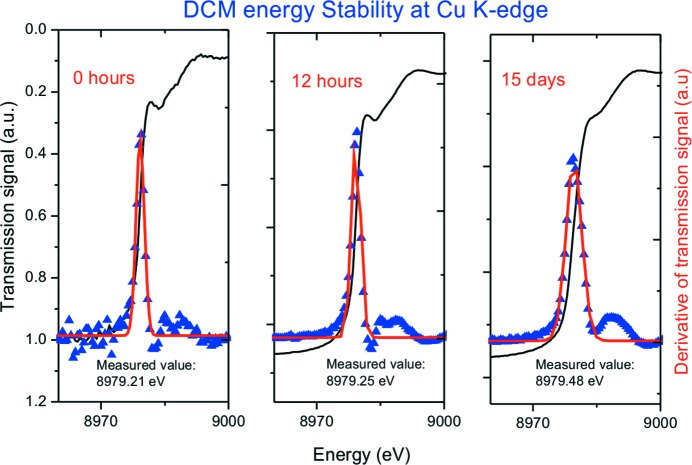
Transmission signal through a Cu foil (black line), the first derivative of the raw data (blue triangles) and a Gaussian fit (red line) of the derivative.

Systematic studies involving repeated absorption edge scans were performed, albeit over relative short periods. Measurements across the Cu *K*-edge taken 12 h apart indicated that the energy was stable to within ±0.02 eV over this period (Fig. 7 ▸). This is better than the proposed stability value of 0.1 eV specified in the Beamline Technical Design Report. The same measurement repeated over two weeks later gave an energy value differing by ∼0.4 eV. This level of energy stability is acceptable, especially considering that during this time there were several beam fills and machine development days.

The energy stability of the instrument depends principally on two parameters: the angle of the beam incident on the first crystal and the variation in the calibration of the angle of the monochromator as it moves. The former can be determined using the two XBPMs where the *Y*-positions (vertical) provide a measure of the angle of the beam and any variation can be characterized over a period of time. During the initial beamline commissioning, we analysed the angular change in the beam using the XBPMs and found a drift of ±1 µrad over a 48 h period. This level of drift is acceptable for the energy range of the B16 beamline. The monochromator Θ-axis is encoded and we have seen no evidence that its repeatability measured during the Factory Acceptance Tests is not being achieved.

### 3.3. Analysis of rocking curves of the DCM   

The B16 directly water-cooled DCM system is characterized by a number of channels drilled near the diffracting surface of the first crystal. Heat transfer studies using FEA were undertaken to optimize the cooling system geometry, to reduce the presence of stress induced by the cooling channels, to obtain a good heat transfer and to remove crystal distortions induced by the beam power load. Experiments were performed to acquire several beam profiles using a Photonic Science ImageStar9000 CCD detector (3056 × 3056 pixels and 31 µm pixel size) to image the transmitted beam under different power load conditions. Data were collected with an unattenuated beam and with different filters inserted upstream and downstream of the DCM to reduce the beam power load and to filter out the first-order reflection contribution. Due to the high intrinsic resolution associated with the high-order harmonics, this arrangement increases the sensitivity of crystal deformation measurements. Images were collected by adjusting the second crystal pitch angle across the double-crystal rocking curve. The measurements were performed at a distance of 27 m from the DCM (47 m from the B16 bending magnet source).

**Figure 8:**
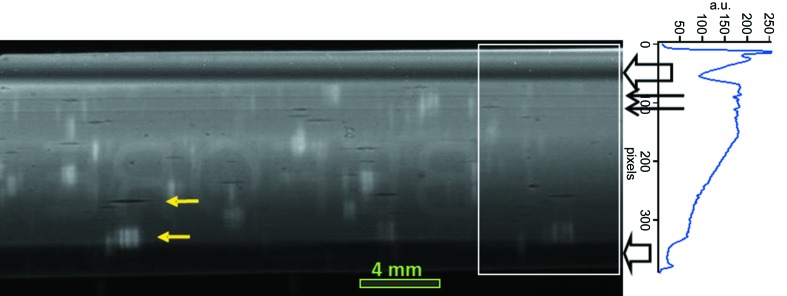
Full crystal image with full unfiltered beam illumination at 15 keV. The pitch on the second crystal was *deliberately* detuned so that all possible crystal defects and mechanical deformations were visible. A 1.4 mm-thick aluminium cover was present on the detector leading to the artefact mirrored letters ‘BEFORE’ being visible in the image centre (these letters are engraved on the detector cover). On the right, the beam intensity profile derived by averaging the intensity in the horizontal direction over the region marked on the image by a white rectangle. At the top and bottom of the image, effects of the clamping system (wide dark bands indicated by large arrows on the right of the image) are clearly visible and, at the top, two lines corresponding to the first pair of cooling channels (thin arrows) are also observed. Small horizontal marks (cavities) and associated bright areas (due to crystal bulk defects), marked by yellow arrows, are also visible and the latter can be seen when the crystal is strongly detuned from its exact Bragg position.

Images of the beam taken under all conditions showed large dark horizontal bands due to deformations induced on the crystal by the mounting system (through holes for clamping) and can be clearly seen in Fig. 8 ▸, an image of the beam taken at 15 keV under full beam illumination with the second crystal strongly detuned with respect to the first crystal. Intensity variations associated with the pair of cooling channels nearest to the clamping holes, visible as thin continuous horizontal lines in the figure, were also observed. The wide bands from the crystal clamping and artefacts corresponding to the cooling channels were present both under full beam power and low power load conditions (after the insertion of a 2 mm-thick Al attenuator upstream of the DCM), indicating the presence of mechanical stress but not a dominant thermal component in the crystal border areas.

The rocking curve measurements were also carried out with the ionization chamber as a detector under different beam power loads in order to evaluate the influence of heat load on crystal performance.

**Figure 9:**
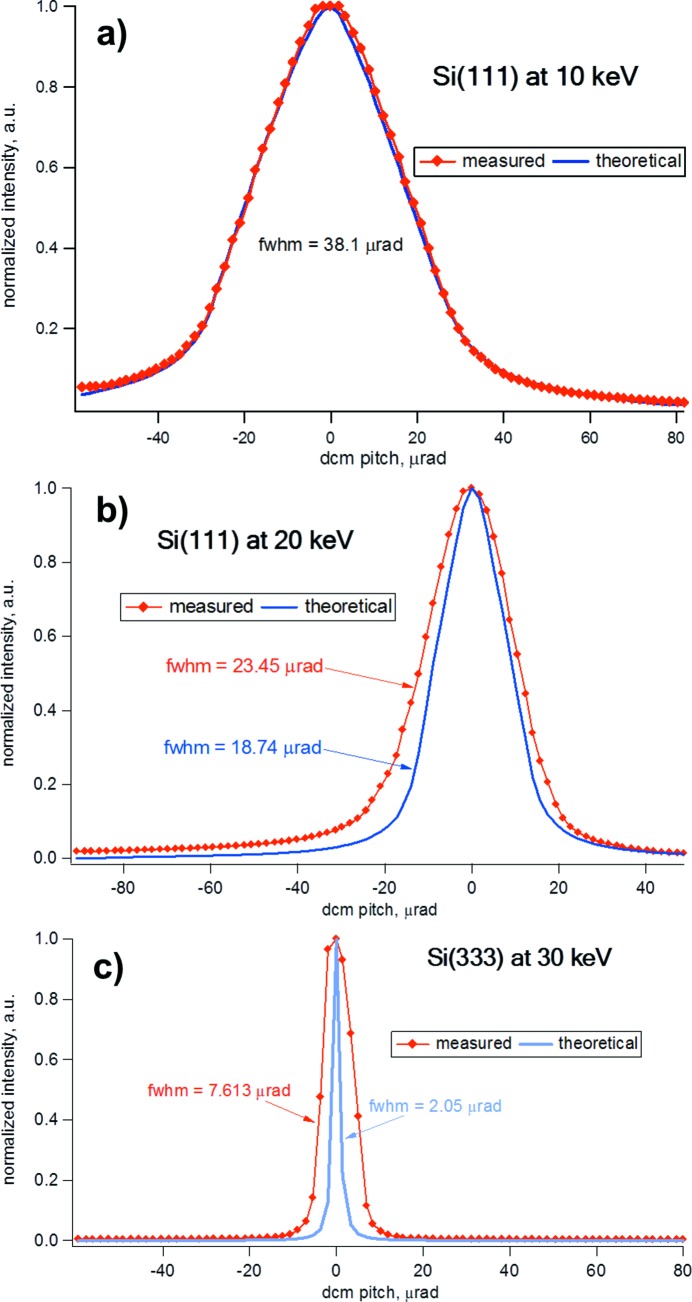
DCM rocking curve profiles comparing experimentally measured (red) and theoretically calculated values (blue) for unperturbed crystals: (*a*) Si(111) reflection with full beam at 10 keV, (*b*) Si(111) reflection with full beam at 20 keV and (*c*) Si(333) reflection with filtered beam at 30 keV.

Fig. 9(*a*) ▸ shows the rocking curve measured with the full beam (full power load) and energy set at 10 keV (with the DLS storage ring operating at the electron beam current of 250 mA) by scanning the pitch of the second DCM crystal. It agrees well with the theoretical rocking curve calculated by self-convolution of a single-crystal rocking curve since the beam undergoes two consecutive reflections from two DCM crystals (*XOP* software package; *X-ray Oriented Programs*, http://www.esrf.eu/Instrumentation/software/data-analysis/xop2.4). The FWHM of the rocking curve was ∼38 µrad and confirms our initial FEA calculations where the thermal load does not affect the DCM performance at this energy.

**Figure 10:**
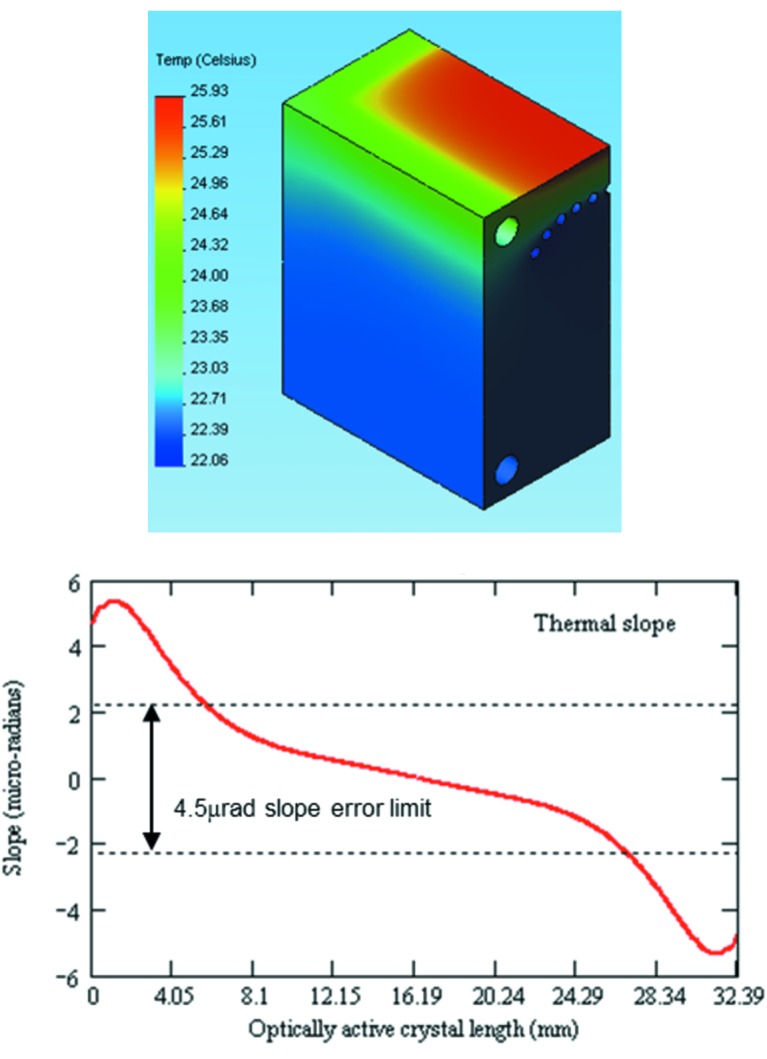
Temperature distribution (top) and thermal slope (bottom) at 20 keV. To achieve an efficient photon flux transmission at the second DCM crystal, the slope error on the first crystal should be a fraction of the rocking curve width, which for Si(111) at 20 keV is ∼14 µrad. Based on an integrated reflectivity of 70%, the allowable maximum slope error was calculated to be 4.5 µrad. About 65% of the optically active beam footprint has been shown to be below the 4.5 µrad slope error limit.

For the Si(111) reflection at 20 keV, with full beam on the first crystal (full crystal illumination with a total 0.25 mrad vertical beam divergence as defined by a wide vertical primary slit aperture), a rocking curve width of 23.45 µrad differed from the theoretical value of 18.74 µrad by ∼4.7 µrad [Fig. 9(*b*) ▸]. This broadening can be compared with the results of the FEA simulations that showed that the crystal tangential deformation profile is within the 4.5 µrad slope error limit (see Fig. 10 ▸ in Appendix *A* [App appa]) over ∼65% of the optically active crystal length when the beam is limited to a 0.16 mrad vertical beam acceptance. This could qualitatively account for the observed difference between the measured and theoretical rocking curve widths.

With the intention of installing the Si(311) channel-cut crystal into the existing DCM mechanism, an analysis of the rocking curve of the Si(333) reflection (third harmonic) of the DCM was performed at 30 keV. The fundamental Si(111) reflection was effectively filtered out by the insertion of 2.5 mm-thick Al plate placed upstream of the DCM. The experimental FWHM of 7.6 µrad was broader than the theoretical width at this energy [Fig. 9(*c*) ▸]. However, the measured line profile does not show any particular asymmetries indicating the complete thermal equilibration of the crystal. Furthermore, under the same geometry, *i.e.* at a Bragg angle of 11.4° which corresponds to an equivalent energy of 19.15 keV for Si(311) crystal pairs, the intrinsic resolution agrees with the FWHM of the measured rocking curve fairly well.

In summary, the crystal cooling system of the DCM copes well with present operating conditions. The clamping and cooling system deformations are visible when imaging the full beam and they are probably mainly due to mechanical stresses induced by the process of manufacturing the water-cooling channels. The measured Si(111) reflection profiles reproduce well the theoretical calculations at low energy, whereas at high energy the difference in the FWHM can be explained taking into consideration the results of FEA simulations.

Due to the absence of an appropriate actuator, the rocking curve measurements with channel-cut crystal were not feasible. However, the measured intensity recorded for the Si(311) channel-cut is a factor of about eight less than the Si(111) DCM, under the same beam conditions and energies of 10 and 20 keV (measurement of absolute flux made with a calibrated photodiode from Physikalisch-Technische Bundesanstalt, Germany), indicating a good performance of the channel-cut crystal under the same heat load. This conclusion can be drawn because, in theory, the intensity ratio of the Si(311) and Si(111) reflections (squared as the beam undergoes two consecutive reflections in the DCM and channel-cut) is (*I*_311_/*I*_111_)^2^ ≃ 0.126 (*PowderCell*, http://www.CCP14.ac.uk/tutorial/powdcell/) and, thus, close to the experimental factor derived above.

## 4. Conclusion   

The double-crystal monochromator has been in operation on the B16 Test beamline for more than 11 years, during which no major problems have arisen with its performance over this long time span. A few modifications and upgrades have been made to the monochromator during the course of its operation. The implementation of the water-pressure pulse reduction system has led to much better beam stability and the addition of a Si(311) channel-cut crystal has extended the beamline working energy range up to ∼45 keV.

The water-cooled monochromator technology is limited in its application to moderate beam power beamlines, such as ones based on bending magnets, whereas for more powerful beamlines built on wigglers or undulator insertion devices cryogenic solutions are more appropriate.

## Figures and Tables

**Figure 11:**
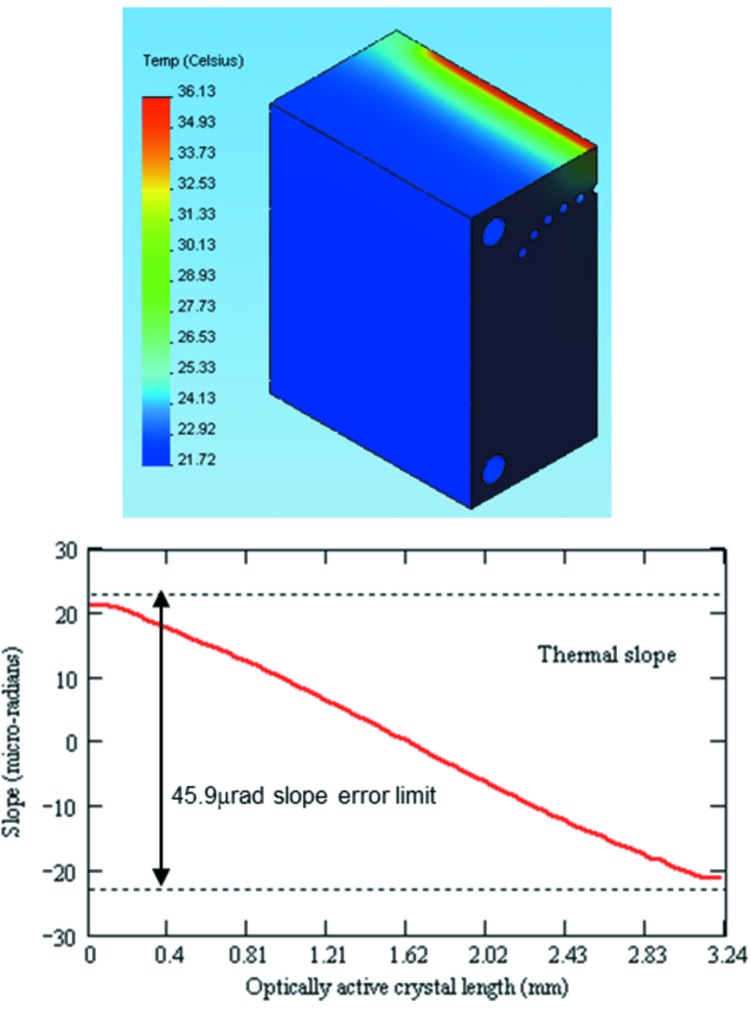
Temperature distribution (top) and thermal slope (bottom) at 2 keV. To achieve an efficient photon flux transmission at the second DCM crystal, the slope error on the first crystal should be a fraction of the rocking curve width, which for Si(111) at 2 keV is 918 µrad. Based on an integrated reflectivity of 99%, the allowable maximum slope error was calculated to be 45.9 µrad. The slope error is acceptable in a sense that there would be no significant transmission intensity losses.

**Table 1:** Summary of monochromator motion parameters

Axis	Range	Step size	Repeatability
Rotary table (Bragg angle)	−0.5 to 85°	0.35 µrad (full step)	<1 µrad
Base height (*Y*) adjustment	±25 mm	0.05 µm	<0.5 µm
X-translation of entire crystal mechanism	0–110 mm	10 µm	10 µm
Second crystal cage: perpendicular (*Y*) adjustment	0–100 mm	1 µm	1 µm
Second crystal cage: coarse pitch	±1°	0.65 µrad (half step)	±1.5 µrad
Second crystal cage: fine pitch (piezo)	±200 µrad	0.05 µrad	<0.1 µrad
Second crystal cage: coarse roll	±1°	0.42 µrad (half step)	±1 µrad

**Table 2:** Slope error limits (µrad)

	Energy (keV)				
Integrated reflectivity (%)	2	5	10	15	20
70	328.0	19.4	9.1	6.0	4.5
80	262.4	12.8	6.0	4.0	3.0
90	183.7	6.3	3.0	2.0	1.5
99	45.9				

## Footnotes

1 The DCM was designed and built by Instrument Design Technology Ltd, in close collaboration with DLS.

## Appendix A FEA studies of the performance of the first Si crystal of the DCM under a specified heat load

For FEA studies (using COSMOSWorks 2006 software) the model for the silicon crystal was reduced to a quarter of the actual size. Element sizing can be characterized as fine to very fine, especially around a heat loaded area and regions considered susceptible to high stress concentrations. For the thermal FEA model, convective cooling was applied to the internal wetted surfaces of the cooling bore holes. The flow rate through the crystal was set above that required to attain turbulent flow (Reynolds number > 2300 for improved heat transfer) to limit the vibrational instability. Based on a flow rate of 3.5 l min^−1^ and using the Dittus–Boelter correlation, a heat transfer coefficient of 1.6 W cm^−2^ K^−1^ was calculated. To apply the thermal load, small areas were projected onto the optical surface and individual heat flux values applied to each area to simulate a Gaussian heat load profile. Based on the DLS bending magnet parameters and a horizontal and vertical acceptance of 3 and 0.16 mrad, respectively, the total incident heat load on the crystal was calculated to be 130 W (*XOP* software package). The results from the thermal model were then loaded into a structural model, with restraints applied to allow free expansion.

The rocking curve width is defined as a function of the wavelength and the energy resolution,

where ω = rocking curve width (radians^2^), *E*_res_ = energy resolution [1.4 × 10^−4^ for Si(111)] and λ = wavelength (Å).

The rocking curve width is the full width at half-maximum (FWHM) value for the curve that defines the peak shape when rocking the crystal in the diffracted beam. Assuming a Gaussian curve form, a relative distribution can be determined using the FWHM value, ω, as given below,

where α is an angular width. Any slope error on the crystal surface will cause a shift in this distribution,

where Δα is the slope error.

The amount of the beam reflected is defined by the region shared by the relative distributions of these two functions. The fraction of the beam reflected can be determined by integrating the shared region for the reflected beam and dividing by the integrated area under the Gaussian distribution with no slope errors. Since the FWHM is a function of energy, then the slope error limit for the crystal will also depend on beam energy. Table 2 ▸ details the slope error limits based on integrated reflectivity.

The results of simulations are shown in Figs. 10 ▸ and 11 ▸ for two extreme energies of 20 and 2 keV, respectively.

Therefore, FEA studies reveal that in the energy range specified for the DCM the angular slope errors on the first crystal are less than or close to the respective theoretical rocking curve widths confirming almost no flux losses.

## References

[bb1] Freund, A. K., Arthur, J. R. & Zhang, L. (1997). *Proc. SPIE*, **3151**, 216–226.

[bb2] Matsushita, T. (1983). *X-ray Monochromators*, in *Handbook on Synchrotron Radiation*, Vol. 1, edited by E. E. Koch. Amsterdam: North-Holland.

[bb3] Sawhney, K. J. S., Dolbnya, I. P., Tiwari, M. K., Alianelli, L., Scott, S. M., Preece, G. M., Pedersen, U. K. & Walton, R. D. A. (2010). *AIP Conf. Proc.* **1234**, 387–390.

[bb4] Schulte-Schrepping, H., Heuer, J. & Hukelmann, B. (1998). *J. Synchrotron Rad.* **5**, 682–684. 10.1107/S090904959701978X 15263618

[bb5] Suortti, P., Fiedler, S., Bravin, A., Brochard, T., Mattenet, M., Renier, M., Spanne, P., Thomlinson, W., Charvet, A. M., Elleaume, H., Schulze-Briese, C. & Thompson, A. C. (2000). *J. Synchrotron Rad.* **7**, 340–347. 10.1107/S0909049500008384 16609218

[bb6] Wahl, R., Shah, R., Jackson, K. & Tonnessen, T. (1992). *Nucl. Instrum. Methods Phys. Res. A*, **318**, 908–913.

[bb7] Zhang, L., Hoszowska, J., Migliore, J.-S., Mocella, V., Ferrero, C. & Freund, A. K. (2001). *Nucl. Instrum. Methods Phys. Res. A*, **467–468**, 409–413.

